# Validity and Reliability of the Portuguese Version of the Nurses’ Professionalism Inventory

**DOI:** 10.3390/nursrep16040117

**Published:** 2026-03-31

**Authors:** Marlene Patrícia Ribeiro, Renata Cristina Gasparino, Olga Maria Pimenta Lopes Ribeiro

**Affiliations:** 1 ICBAS, Abel Salazar Institute of Biomedical Sciences, University of Porto, 4050-313 Porto, Portugal; 2 RISE-Health, Faculty of Medicine, University of Porto, 4200-319 Porto, Portugal; 3 Tâmega and Sousa Local Health Unit, 4560-136 Penafiel, Portugal; 4 School of Nursing, Universidade Estadual de Campinas—Unicamp, Campinas 13083-887, São Paulo, Brazil; grenata@unicamp.br; 5 RISE-Health, Nursing School, University of Porto, Rua Dr. António Bernardino de Almeida 830/844/856, 4200-072 Porto, Portugal; olgaribeiro@esenf.pt

**Keywords:** nurse managers, nursing, professionalism, psychometrics, validation study

## Abstract

**Background/Objectives**: Professionalism reflects an individual’s connection, identity, and dedication to their profession. In nursing, it is associated with quality of care and professional respect, making its assessment essential for workforce development and management. However, valid and reliable instruments are needed to measure this construct across cultural contexts. Therefore, this study aims to evaluate the validity and reliability of the Portuguese version of the Nurses’ Professionalism Inventory (NPI). **Methods**: This methodological study used cross-sectional data collected from November 2024 to January 2025 in northern Portugal. Data were gathered from a convenience sample of 684 nurses who completed a sociodemographic questionnaire, the Portuguese NPI, the Conditions of Work Effectiveness Questionnaire II (CWEQ-II), and the Team Psychological Safety (TPS) scale. Confirmatory Factor Analysis (CFA) was conducted. Factor loadings and Average Variance Extracted (AVE) were used to assess validity. Internal consistency was evaluated using Composite Reliability, McDonald’s omega, and Cronbach’s alpha. Convergent validity was examined using Spearman correlations among NPI subscales, CWEQ-II dimensions, and TPS. **Results**: The Portuguese version of NPI preserved the original five-factor structure. The model showed acceptable fit indices (TLI = 0.90; CFI: 0.91; RMSEA = 0.10; SRMR = 0.08). All items had factor loadings above 0.50, except item 18 (0.42), which did not load significantly on any other factor; therefore, it was removed. This improved the AVE of the Professional Attitude subscale. The overall internal consistency was satisfactory, with all reliability coefficients ranging between 0.73 and 0.99. The correlations among the NPI subscales, CWEQ-II dimensions, and TPS were positive and statistically significant. **Conclusions**: This study demonstrates adequate measurement properties of the Portuguese version of NPI, supporting its use as a valid and reliable instrument.

## 1. Introduction

Florence Nightingale is widely recognised as a foundational figure in modern nursing, having contributed to the professionalisation and scientific development of the discipline [1,2]. Since then, nursing professionalism has significantly developed and is now seen as a complex and multidimensional concept that reflects nurses’ attitudes, behaviours, and professional values in contemporary systems [3,4].

However, over time, nursing professionalism has evolved and improved, and is recognised as an evolutionary and multidimensional concept [3,4]. It reflects nurses’ attitudes and behaviours towards their work [2] and involves delivering excellent care grounded in core values such as integrity, responsibility, and respect [5].

Concept analyses emphasise cognitive, affective, and psychomotor dimensions, covering professional knowledge, values and identity, and clinical competence, which support professional identity, ethical behaviour, and adherence to standards of practice [4]. Other key aspects identified include the effects of professionalisation in nursing, which encompass improved quality of care, patient and team satisfaction, and greater professional authority and decision-making power, all of which are connected to autonomy and professional empowerment [3,4,6].

Professionalism in nursing can still be regarded as a combination of specialised and complex knowledge and is often linked to specific actions such as participation in scientific activities and engagement with professional literature [3].

Recent studies emphasise the importance of assessing nurses’ professionalism and implementing targeted interventions to improve it, considering its significance for positive outcomes for nurses, the nursing profession, patients, and organisations [7,8]. In this context, professionalism in nursing has been linked to organisational and psychological factors, such as structural empowerment and psychological safety, which influence behaviours, decision-making, and nurses’ engagement in professional practice. Collectively, they support the development of professional behaviours, fostering autonomy, confidence, and active participation in care processes, which are essential components of nurses’ professionalism [7,8].

Despite the importance of professionalism in nursing, in Portugal, no validated instrument exists to accurately and consistently assess it, which has limited scientific research and management investment in this area.

At present, it appears that in the Portuguese context, aspects related to professionalism, such as skills, ethical values, professional conduct, autonomy, empowerment, or the process of constructing the professional identity of nurses are analysed, through specific instruments [9,10] or dimensions or subscales of more comprehensive instruments, such as autonomous practices that can be analysed through the Scale for the Environment Evaluation of Professional Nursing Practice (SEE-NP) [11]. However, no instrument exists to assess this specific construct in this context.

Two recent literature reviews identified instruments used to assess nurses’ professionalism [12,13]. In both cases, the Nurses’ Professionalism Inventory (NPI) [14] was identified as a reliable tool, showing a high degree of adjustment for each of its factors, with almost all measurement properties adequately reported [12,13,14].

Although the NPI does not follow a specific theoretical model, such as those proposed by Hall [15] or Miller [16], these frameworks have historically contributed to conceptualising professionalism as a combination of values, behaviours, and professional identity. Contemporary approaches, however, view nurses’ professionalism as a dynamic, context-dependent construct shaped by the interaction between individual attributes, professional identity development, and organisational environments [17,18]. A recent model proposed by Parastesh et al. emphasises professionalism as an integration of core values and competencies, including ethical practice, accountability, autonomy, and continuous development, alongside relational and reflexive aspects such as professional interactions and self-awareness [17]. Another contemporary model proposed by Cao et al. further highlights fundamental values such as altruism, caring, dedication, and professional competence as central to nursing professionalism [18]. In this sense, the NPI reflects a more recent and integrative perspective, incorporating dimensions aligned with current nursing practice, including research and innovation, as well as other elements previously included in other instruments, such as ethical conduct, accountability, autonomy, ongoing training, and professional affiliation [13,14].

The NPI was developed by Ichikawa et al. [14] based on an extensive literature review of the conceptual structure of nursing professionalism as conceptualised by the researchers, which encompassed “Accountability”, “Self-improvement”, “Professional Attitude”, “Advancement of the Nursing Profession”, and “Professional Membership”. This same structure gave rise to the instrument’s five subscales, which contain a total of 28 items [14]. Currently, the NPI has enabled the evolution of knowledge and investment in this area, not only in Japan but also in Slovakia [14,19].

Thus, although classical models were essential in shaping the concept of professionalism in nursing [15,16], they reflect earlier stages of the profession’s development and do not fully represent its current evolution in Portugal [20].

In recent decades, Portuguese nursing has experienced a major transformation, characterised by discussions on expanding advanced nursing practices, higher levels of training, and increased involvement in research, innovation, and organisational development [20,21]. This transformation has been supported by the profession’s regulatory framework since the establishment of the Portuguese Order of Nurses in 1998. Additionally, other Portuguese professional nursing associations have been formed over the past two decades, contributing to this progression towards a more contemporary model while still upholding the profession’s fundamental and structuring values [20,21].

These nuances are reflected in the NPI, which closely aligns with these contemporary demands by incorporating subscales such as the Advancement of the Nursing Profession, Professional Attitude, and Self-Improvement, all of which strongly resonate with current professional expectations in the Portuguese context.

Furthermore, a recent cross-cultural adaptation and content validation of the NPI in the Portuguese context confirmed its appropriateness and conceptual relevance, reinforcing its importance as a coherent and comprehensive instrument for assessing professionalism among nurses in this country [22]. In addition, it provided a solid basis for the subsequent evaluation of measurement properties.

The validation of instruments measuring nursing professionalism is of international relevance, as it enables cross-cultural comparisons, supports the development of global nursing standards, and promotes research and practice across different healthcare systems [12,13]. Ensuring that such instruments demonstrate robust measurement properties in diverse contexts is essential to strengthen their applicability and comparability at an international level.

Building on this background, the present study aims to analyse the validity and reliability of the Portuguese version of the NPI, providing a robust and valuable tool for research, training, and management in nursing.

## 2. Materials and Methods

### 2.1. Design and Setting

This study employed a methodological and cross-sectional design to assess the validity and reliability of the Portuguese version of the NPI, according to the checklist criteria of the Consensus-based Standards for the selection of health Measurement Instruments (COSMIN) [23].

The research was conducted from November 2024 to January 2025 in one of the largest university hospitals in the country, located in the northern region of Portugal.

This hospital serves a broad population and is also a referral centre for many medical and surgical specialities in the northern part of the country. It has around 1100 beds and employs 6675 professionals from various parts of the country, including approximately 2580 nurses.

### 2.2. Participants and Sample

The sampling technique used was non-probabilistic convenience sampling. The criterion established for factor analysis and instrument validation, which requires 5 to 10 participants for each item of the instrument, was considered [24,25]. This determined a minimum sample size of 140 participants, as the Portuguese version of the NPI tested contains 28 items.

All nurses working in the hospital with at least six months of professional experience in the setting could be included, regardless of employment relationship or professional category.

Nurses absent due to leave, vacation, or medical certificates of inability or impediment to work during the data collection period were not included.

### 2.3. Data Collection Instrument

The data collection instrument consisted of two parts: the first part consisted of a questionnaire for sociodemographic, professional, and academic characterisation, and the second part contained the version of the NPI cross-culturally adapted for the Portuguese context [22], the Portuguese versions of the Conditions of Work Effectiveness Questionnaire II [26], and Team Psychological Safety [27].

#### 2.3.1. Sociodemographic, Professional, and Academic Characterisation

The first part of the questionnaire consisted of closed-ended questions to analyse the sample’s sociodemographic, professional, and academic characteristics. This form was developed by the research team, and the selection of variables was based on factors associated with nurses’ professionalism [7,14], as well as the objectives of the present study. All variables were chosen to minimise respondent burden while ensuring the collection of relevant contextual data.

For personal characterisation variables, age and gender were recorded. Regarding professional characterisation variables, the unit and current work setting, total professional experience, and, in the current context, nursing speciality and professional category were documented. Concerning academic characterisation, only academic qualifications were recorded.

#### 2.3.2. Nurses’ Professionalism Inventory (NPI)

The NPI is an instrument that allows for assessing nurses’ professionalism levels in different contexts. It was initially constructed in the Japanese context by Ichikawa et al. (2020), based on a literature review that focused on a conceptual framework encompassing “accountability”, “self-improvement”, “professional attitude”, “advancement of the nursing profession”, and “professional membership” [14].

The development of the instrument resulted in the respective five subscales, comprising a total of 28 items: Accountability (8 items; 1, 2, 3, 4, 5, 6, 7, and 8), Self-improvement (8 items: 9, 10, 11, 12, 13, 14, 15, and 16), Professional Attitude (5 items: 17, 18, 19, 20, and 21), Advancement of the Nursing Profession (4 items: 22, 23, 24, and 25), and Professional Membership (3 items: 26, 27, and 28) [14].

The application of the instrument involves each participant indicating to what extent the items reflect their awareness and behaviours related to nursing on a 6-point Likert scale, ranging from “1 = Does not reflect at all” to “6 = Reflects completely” [14].

Nurses’ Professionalism Inventory subscale scores are calculated by summing the scores of the constituent items for each subscale, with higher scores indicating greater professionalism among nurses [14].

In the validation study of the original version, the NPI underwent both exploratory and confirmatory factor analyses. Internal consistency was high, with Cronbach’s alpha coefficients ranging from 0.83 to 0.90 for the subscales and reaching 0.95 for the overall scale. Confirmatory factor analysis supported a good fit for the five-factor structure. Test–retest reliability was reported as excellent for the overall scale (ICC = 0.94; 95% CI: 0.92–0.95) and acceptable to excellent across subscales (ICC = 0.73–0.88), indicating strong temporal stability of the instrument. Concurrent validity was supported by significant correlations between the NPI (total and subscales) and global measures of professionalism [14].

#### 2.3.3. Conditions of Work Effectiveness Questionnaire II (CWEQ-II)

The Conditions of Work Effectiveness Questionnaire II (CWEQ-II) is an instrument for assessing structural empowerment. In this study, structural empowerment was employed because it can significantly influence nurses’ professionalism, as it affects how they perceive their support, value, and ability to provide quality care within healthcare organisations. According to Kanter’s model, structural empowerment refers to access to resources, information, support, and opportunities within the work environment, which are essential elements for nurses’ professional development and autonomy [28].

The CWEQ-II was initially developed by Laschinger et al. [29] and adapted and validated for the Portuguese cultural context by Teixeira et al. [26]. The instrument contains a total of 19 items distributed across six dimensions: Opportunity (3 items), Information (3 items), Support (3 items), Resources (3 items), Formal Power (3 items), and Informal Power (4 items) [26].

When applying the instrument, a 5-point Likert scale is used that is appropriate for each dimension. Thus, for the dimensions of Opportunity, Formal Power, and Informal Power, the scale ranges from 1 (None) to 5 (Many); in the Information dimension, it ranges from 1 (No knowledge) to 5 (A lot of knowledge), and for the Support and Resources dimensions, it ranges from 1 (None) to 5 (A lot). The score is obtained by adding the averages of the six dimensions, resulting in a total score ranging from 6 to 30, with higher values indicating greater structural empowerment [26].

In the cross-cultural adaptation and validation study of the CWEQ-II for the Portuguese context, the instrument demonstrated strong psychometric properties. The overall Cronbach’s alpha coefficient was 0.911, with individual dimension values ranging from 0.678 to 0.889 (Opportunity = 0.854, Resources = 0.797, Information = 0.859, Support = 0.889, Formal Power = 0.811, and Informal Power = 0.678) [26]

In the current study, all dimensions showed Cronbach’s alpha values above 0.70: Opportunity (0.84), Information (0.91), Support (0.92), Resources (0.84), Formal Power (0.80), and Informal Power (0.75).

#### 2.3.4. Team Psychological Safety (TPS)

Team Psychological Safety scale (TPS) is a tool that enables the assessment of psychological safety within teams.

In this study, psychological safety within nursing teams is examined because it can be considered a key factor in developing professionalism.

This construct refers to the belief held by team members that the team provides a safe environment in which they can take risks [30]. It means that professionals perceive that they can express ideas, concerns, questions, or mistakes without fear of reprisal, judgment, or humiliation. When healthcare professionals feel psychologically safe, they are more willing to collaborate, learn from one another, and take on responsibilities with confidence. Such an environment encourages ethical behaviour, commitment to the profession, autonomy, and initiative, all of which are core elements of professionalism [30,31].

This instrument was initially constructed by Amy Edmondson [30] and cross-culturally adapted and validated for the Portuguese context by Ferreira [27].

It consists of seven items, three of which are reversed (items 1, 3, and 5), with a Likert-type response scale ranging from 1 (Does not apply) to 7 (Completely applies). The result is interpreted based on the average scores of all items, with higher scores indicating greater team psychological safety [27].

In the cross-cultural adaptation and validation of the TPS scale for the Portuguese context, the instrument showed a Cronbach’s alpha of 0.70 [27], whereas in the present study, it was 0.64.

### 2.4. Data Collection Procedures

In each department of the hospital, the nurse manager or another designated nurse was requested to collaborate on verifying the inclusion and exclusion criteria for potential participants, distributing and collecting questionnaires, and obtaining the Informed Consent Form.

The appropriate number of questionnaires and Informed Consent Forms were made available in paper format. The questionnaires were delivered, collected in a sealed envelope, and adequately separated from the original Informed Consent Form. For each service, two dates were scheduled for clarification of any possible doubts or provision of additional information, as well as intermediate and final collection of questionnaires.

### 2.5. Statistical Analysis

The data were organised in Microsoft Excel^®^ (Office 365) version 2501. A preliminary analysis was conducted to identify and correct any potential input errors. Simple descriptive statistical methods were used to analyse the sample characterisation variables.

Structural construct validity was evaluated using confirmatory factor analysis with the Weighted Least Squares Means and Variances (WLSMV) estimation method. This is a robust estimator suitable for analysing categorical data, such as Likert-scale responses, in models like confirmatory factor analysis. The goodness of fit of the factor model was assessed using the Comparative Fit Index (CFI), Tucker–Lewis Index (TLI), Root Mean Square Error of Approximation (RMSEA), and Standardized Root Mean Square Residual (SRMR). Values of CFI and TLI greater than 0.90, RMSEA less than 0.10 (Confidence Interval [CI] = 90%), SRMR less than 0.08, and factor loadings (λ) of 0.50 or higher were considered acceptable [24,32].

The Average Variance Extracted (AVE) for each factor was evaluated to analyse the proportion of variance in the items that is explained by their respective factor. AVE values greater than 0.50 indicate satisfactory model convergence [24].

Composite reliability, McDonald’s omega, and Cronbach’s alpha were calculated to assess the instrument’s internal consistency, with values above 0.70 deemed satisfactory [24,33].

Spearman’s correlation test between the NPI subscales, the CWEQ-II dimensions, and the TPS was used to assess convergent construct validity, as it does not assume that the data follow a normal distribution and evaluates the strength and direction of the relationship between two variables. This coefficient can range from −1 to 1. Values closer to −1 indicate a negative relationship, while those nearer to 1 suggest a positive one. Values around 0 imply no relationship [34]. Correlation coefficients between 0.1 and 0.29 are considered weak, between 0.30 and 0.49 are moderate, and values of 0.50 or higher are deemed strong [35].

The hypothesis that the greater the professionalism, the greater the structural empowerment and psychological safety of nurses was tested.

For each test performed, a significance level of 5% (α = 0.05) was adopted, corresponding to a commonly used confidence level of 95%. When *p* < α, the null hypothesis was rejected, indicating a statistically significant result at the adopted significance level.

All analyses were performed using R-Project version 4.3.3.

### 2.6. Ethical Considerations

The cross-cultural adaptation and validation study of the NPI for the Portuguese context was approved and has been monitored by the author who developed the original instrument. Furthermore, the use of the Portuguese versions of the Conditions of Work Effectiveness Questionnaire II [26] and Team Psychological Safety [27] instruments was approved by the respective authors.

All potential participants received a Participant Information Letter that conveyed the study’s objectives and purpose, methods, risks, and benefits, as well as the voluntary nature of participation and the right to withdraw at any time without incurring any future harm or loss. The institution’s Free and Informed Consent Model was utilised. No data that could identify the participants was collected; furthermore, all collected data were coded, anonymised, and securely stored in password-protected files with restricted access to the research team.

This study protocol was analysed, received a favourable opinion from the Health Ethics Committee and the Data Protection Officer, and was authorised by the hospital’s Board of Directors (n. 334/2024). It was conducted in accordance with the Declaration of Helsinki.

## 3. Results

### 3.1. Sample Description

There was a response rate of 58.25%. After excluding incomplete questionnaires, the final sample consisted of 684 participants.

The sample consisted predominantly of female participants (*n* = 553; 80.8%), with a mean age of 40.3 years (SD = 9.4). Most participants held a bachelor’s degree in nursing (*n* = 576; 84.2%) and were in the professional category of registered nurse (*n* = 526; 76.9%). However, 251 participants (36.7%) reported holding the title of specialist nurse, with rehabilitation nursing and medical–surgical nursing being the most represented specialities, with 78 (31.1%) and 56 participants (22.3%), respectively. The sample had an average of 17.0 years (SD = 9.3) of professional nursing experience and a mean of 11.0 years (SD = 9.0) of experience in their current workplace. Regarding the work setting, most participants were from medical (*n* = 223; 32.6%) and surgical departments (*n* = 203; 29.7%). Table 1 summarises the participants’ sociodemographic, professional, and academic characteristics.

### 3.2. Validity and Reliability

The initial results of the model fit assessment revealed that the CFI and TLI were 0.91 and 0.90, respectively. The RMSEA reached 0.09 (90% CI [0.09, 0.10]), while the SRMR was 0.07.

The AVE was below the recommended threshold only for the subscales of Accountability (0.48) and Professional Attitude (0.46) (Table 2). The internal consistency values for each of the subscales of the Portuguese version of the NPI were higher than 0.7 (Table 2).

Upon reviewing the items within these subscales, only item 18 (Professional Attitude Subscale) presented a factor loading below 0.5 (factor loading of item 18 = 0.42). Additionally, the item did not load significantly on any other factor and was therefore removed from the model.

Following its removal, the final factorial model demonstrated acceptable fit indices, with a CFI of 0.91 and a TLI of 0.90. The RMSEA was 0.10 (90% CI [0.09, 0.10]), and the SRMR was 0.08. The AVE for the Professional Attitude subscale increased to 0.52. The AVE values and the internal consistency of the final model from the Portuguese version of the NPI subscales are presented in Table 3.

All the factor loadings of the items in their respective constructs in the final model are presented in Table 4.

**Figure 1:**
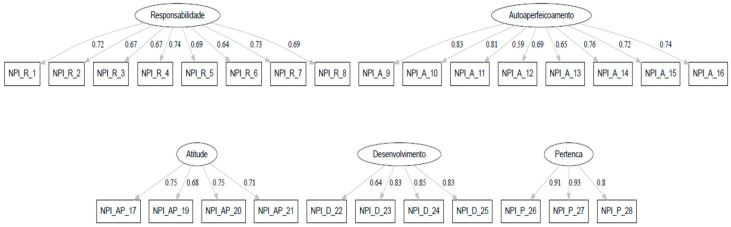
Confirmatory factor analysis (CFA) of the final five-factor model of the NPI. Standardised factor loadings between latent variables and observed items are presented.

The final confirmatory factor analysis (CFA) model is presented in Figure 1. All items loaded on their respective factors, with standardised factor loadings ranging from 0.59 to 0.93.

The correlations between the NPI subscales, the dimensions of the CWEQ-II, and the TPS were all positive and statistically significant (*p* < 0.05). The results of the Spearman correlation tests are presented in Table 5.

## 4. Discussion

To ensure the success and quality of the previously conducted cross-cultural adaptation process of the NPI for the Portuguese population [22], this study assessed construct validity—structural validity and convergent validity, through hypothesis testing—and reliability.

The study involved 684 nurses from a university hospital in northern Portugal, one of the country’s leading hospitals, ensuring diversity in participants’ backgrounds, departmental context, and experiences.

The sociodemographic, professional, and academic characteristics of this sample aligned with national data on Portuguese nurses, supporting its representativeness. Specifically, 80.8% of participants were female and 19.2% male, compared to approximately 82.7% and 17.3% reported nationally, with data from 2023, by the National Institute of Statistics [36].

Regarding age, the sample in this study had a mean age of 40.3 (SD = 9.4) years, corresponding to the age range of the most prevalent Portuguese nurses in 2024, as reported by the Order of Nurses [37].

In terms of professional category, 76.9% were nurses and 22.1% were specialist nurses, while in Portuguese hospitals, 78.2% were nurses and 21.8% were specialist nurses [36]. Regarding specialisation, Rehabilitation Nursing and Medical–Surgical Nursing were the most common specialities in this study, and are also the most common in Portugal [36,37]. These data indicate a sample with a profile compatible with the nursing population in Portugal.

Confirmatory factor analysis is a theory-driven technique that requires a previously tested model to determine whether the observed data fit the proposed model. Therefore, since the original instrument has already been subjected to an exploratory factor analysis [14], a confirmatory factor analysis was used with the primary objective of assessing the validity of the Portuguese version of the NPI. The results of this study confirm that the original version of the NPI presents a consistent five-factor structure, which is consistent with the original validation study conducted in Japan, which also corroborated a five-factor model with adequate fit indices and strong internal consistency [14]. Similar multidimensional structures have been reported in other studies assessing professionalism in nursing, reinforcing the conceptualisation of professionalism as a multidimensional construct across different cultural contexts [13]. These findings suggest that the Portuguese version of the NPI assesses nurses’ professionalism as a multidimensional construct, characterised by five interconnected domains: Accountability, Self-improvement, Professional Attitude, Advancement of the Nursing Profession, and Professional Membership. The instrument measures both behavioural and attitudinal elements of professionalism, reflecting nurses’ engagement with ethical practice, ongoing development, professional relationships, and organisational involvement.

In the first model tested, the confirmatory factor analysis revealed that the AVEs of the Accountability and Professional Attitude subscales were lower than recommended, indicating a weakness in the correlation of the items within their respective constructs. In these cases, the literature recommends eliminating items with lower factor loadings [38].

Therefore, considering that the factor loading of item 18 (“I maintain a certain distance to preserve an objective point of view in relation to others”) was lower than the defined value (factor loading of item 18 = 0.42), this item has been excluded. This exclusion may be related to some difficulty in understanding the content item, given its subjectivity in the Portuguese cultural context, as it presented a factorial load of 0.71 in the original context [14]. The wording of the item in Portuguese does not specify what the distancing refers to, nor the other aspects, which may make it difficult for respondents to understand, leading to more neutral answers.

In this sequence, although the item had not been removed earlier, it had already been modified to improve participants’ understanding. In a previous methodological study on cross-cultural adaptation and content validation of the NPI for the Portuguese context [22], during the first round of expert review, item 18 received a Content Validity Index (CVI) of 0.89, slightly below the accepted threshold of 0.90. This was based on evaluations assessing idiomatic equivalence and the item’s relevance for the dimension being studied in the target context. Furthermore, changes to the wording were suggested without altering the content, which could already indicate difficulties in understanding the item, even though the authors did not report any recommended adaptations during the pre-test [22].

Additionally, the item conditioned the AVE from the Professional Attitude subscale, which improved after the item was removed (AVE Professional Attitude: 0.46 to 0.52). This subscale includes items that reflect proactive decision-making in the best interests of patients, by nurses [14] and it was composed of items 17 (“I maintain a long-term perspective to support the lives of patients”), 19 (“My viewpoints are not clouded by customs, helping me to support the organisation and those in it”), 20 (“I work with the families and those engaged in health care and medicine as an advocate for my patients”) and 21 (“I provide patients and their families with support based on their physical and mental needs at the time”). The maintenance of these four items in the Professional Attitude subscale of the Portuguese version of the NPI aligns with the principles of professional practice outlined in the Portuguese Regulation of Professional Practice for Nurses [39]. This regulatory framework highlights holistic care that addresses patients’ physical and psychological needs, promotes interprofessional collaboration on shared care objectives, and underscores the nurse’s role in organisational improvement.

These results support excluding item 18 from the Portuguese version of the NPI. The low factor loading, evidence of cultural and interpretative ambiguity, and its negative impact on the AVE of the Professional Attitude subscale demonstrate that the item does not function properly within the Portuguese context. Removing it improves metric robustness and conceptual clarity, ensuring that the Professional Attitude subscale more accurately reflects nurses’ proactive and patient-centred decision-making behaviours, as originally intended.

Regarding the model fit, although the final factorial model demonstrated acceptable fit indices (CFI = 0.91; TLI = 0.90; SRMR = 0.08), the RMSEA value of 0.10 (90% CI [0.09–0.10]) is at the upper limit of acceptability [24,32].

Despite this, the combination of appropriate values for CFI, TLI, and SRMR, together with satisfactory internal consistency and an improved AVE after excluding item 18, which had a low factor loading, supports the overall adequacy of the factorial structure. Therefore, although the RMSEA suggests a marginal fit, values around 0.10 have been reported in models with similar characteristics, particularly in studies involving complex constructs and a limited number of indicators per factor. In this context, the RMSEA should be interpreted with caution, as it may overestimate model misfit. However, its impact on the overall interpretation of the model is limited, and the convergence of multiple fit indices and reliability evidence indicates that the model is acceptable for measuring the intended constructs. This is consistent with recommendations that RMSEA should be interpreted alongside other fit indices rather than in isolation. Furthermore, the tested model achieved all indicators, meeting the required standards and aligning with the model of the original instrument [14,24,32].

Regarding the Accountability subscale, when analysing the factor loadings of the items that comprise it, it was observed that all were higher than the values recommended by the literature [24,32]. Continuing with the evaluations, it was possible to note that in all the tests used to analyse internal consistency, the values achieved were also higher than those recommended by the literature [24,33], and, therefore, it was decided not to exclude any items.

It is essential that an instrument consistently measures the intended construct, as determined by reliability analysis. The reliability of the Portuguese version of the NPI was assessed using Composite Reliability, McDonald’s omega, and Cronbach’s alpha. The results indicated ranged from 0.73 to 0.99 for all five factors, indicating high reliability and identical to that of the original version [14,24,33]. Although the McDonald’s omega value is nearly perfect for the Self-improvement subscale (0.99), this reflects consistency of the items content with the concept, rather than redundancy between them, as the eight items of this subscale individually cover: involvement in research; sharing research findings with others; receiving feedback from colleagues and other health professionals; career objectives; professional relationships; consumption of current scientific literature; and the use of conceptual models and nursing theories. So, the content of each item reflects conceptually distinct aspects of professional self-improvement. This supports the breadth of the subscale’s content, indicating that the high reliability is not due to item duplication but rather to the consistency of the construct of related dimensions.

These results are consistent with previous studies that evaluated professionalism instruments in nursing, which generally reported acceptable to high levels of internal consistency and construct validity, although variability in psychometric performance was observed across different contexts [13]. In particular, the original validation study of the NPI showed strong reliability coefficients and appropriate factorial validity, confirming its robustness as a measurement instrument [14].

Thus, after all the analysis of the final tested model, the Portuguese version of the NPI was composed of 27 items distributed across five subscales: Accountability (8 items: 1, 2, 3, 4, 5, 6, 7, 8), Self-improvement (8 items: 9, 10, 11, 12, 13, 14, 15, 16), Professional Attitude (4 items: 17, 19, 20, 21), Advancement of the Nursing Profession (4 items: 22, 23, 24, 25), and Professional Membership (3 items: 26, 27, 28).

The changes made to the number of items in the same instrument aim to stabilise its measurement properties while simultaneously reflecting cultural aspects that differ between countries [40].

The convergent validity of the Portuguese version of the NPI with 27 items was assessed by analysing the correlations between its subscale scores and variables theoretically associated with the same construct, specifically the dimensions of the CWEQ-II and the TPS. The results showed positive and statistically significant correlations across all combinations, indicating an association between the constructs. However, the strength of these correlations was relatively weak and should be interpreted cautiously, as it suggests that while the constructs are theoretically related, they remain conceptually distinct. In terms of convergent validity, moderate correlations are generally expected when comparing related but not identical constructs. Therefore, these findings offer partial support for convergent validity, showing that the constructs are related but not overlapping. Additionally, the internal consistency of the TPS in this study was slightly lower (*α* = 0.64) than in the validation study conducted in the Portuguese context [27]. Although this value is borderline acceptable, it may reflect contextual or sample-specific factors, such as the interpretation of an item, and should be considered when interpreting the correlations, as lower reliability could weaken the observed associations.

In addition to confirming convergent validity, the associations between the NPI subscales, the dimensions of CWEQ-II, and TPS have implications for nursing management and education. These results suggest that higher levels of professionalism tend to emerge in work environments where nurses feel psychologically safe and empowered, having access to opportunities, information, support, resources, and both formal and informal power. Moreover, a more detailed analysis is possible; for example, the Self-Improvement subscale showed its strongest correlations with Informal Power (r = 0.299; *p* < 0.0001) and Information (r = 0.271; *p* < 0.0001) dimensions, indicating that nurses who invest more in continuous development also perceive themselves as better connected across organisational levels and more informed about workplace processes and decision-making [41]. This emphasises the potential usefulness of applying the NPI alongside organisational measures to guide staff development, inform targeted training, and support leadership and career advancement initiatives.

Considering existing theoretical views on nurses’ professionalism and the development of professional identity, the multidimensional structure identified in this study aligns with models that see professionalism as a combination of values, behaviours, and professional identity, shaped by experience and organisational context. Furthermore, the connection between professionalism and organisational factors such as structural empowerment and psychological safety supports existing research emphasising the importance of leadership and the nursing practice environment in shaping professional nursing conduct. Similar findings have been reported in international studies examining nurses’ professionalism and related factors, highlighting the significance of these dimensions across different health systems [7,8,12].

Furthermore, some studies have consistently demonstrated that the assessment tools for professionalism in nursing tend to exhibit multidimensional structures that vary across different cultural contexts, also reflecting contextual specificities. These studies highlight that dimensions such as professional development, engagement with research, and organisational participation are increasingly recognised as central components of contemporary nursing professionalism. In this regard, the findings of the present study align with international evidence, supporting the robustness of the NPI structure and reinforcing its adaptability to the Portuguese context [7,8,12].

In addition to practical applications, this study also presents important theoretical and research implications. The validation of the Portuguese version of the NPI contributes to the advancement of knowledge about professionalism in nursing, supporting its conceptualisation and providing a foundation for future research exploring the relationships between professionalism, the development of professional identity, and organisational and leadership factors in different contexts. The availability of a validated instrument also allows for cross-cultural comparisons and supports the development of more robust evidence on professionalism in nursing at an international level.

Regarding the implications of this study, the availability of a validated and reliable instrument to measure nurses’ professionalism in the Portuguese context enables nurse managers to systematically assess and monitor their team members’ professionalism. Additionally, because it is divided into five subscales, it enables the identification of strengths and weaknesses, thereby informing the setting of priorities and targeted interventions. Content for ongoing training or workshops can be based on the lowest scores within the subscales, such as professional ethics, research, and innovation. For instance, low scores on the Professional Attitude subscale may indicate a need for workshops on ethical decision-making or communication, whereas lower scores on the Self-improvement subscale may inform the development of training initiatives focused on research utilisation, innovation, or participation in organisational improvement projects. In practice, the NPI can assist managers in determining priorities for continuing professional development, guiding the design of targeted educational programmes, mentorship strategies, and clinical supervision plans.

Additionally, as a contribution to nursing management, the importance of assessing nurses’ professionalism is emphasised as an indicator that complements the standard performance appraisal metrics. Moreover, integrating NPI results into team development activities can help foster a culture of professionalism within healthcare organisations and yield positive outcomes for both professionals and patients.

At the policy level, the NPI provides opportunities for integration into institutional accreditation processes, competency frameworks, and nurses’ workforce development policies. By regularly monitoring professionalism, institutions can align professional development strategies with national nursing standards and quality-of-care indicators, reinforcing professionalism as a core organisational priority and a driver of safe and effective practice.

Finally, for decision-makers, the overall levels of professionalism among nurses can be used to promote appreciation and recognition of the profession in the Portuguese context.

### Limitations of the Study

The Portuguese version of the NPI underwent a comprehensive evaluation of its measurement properties. However, the study had several limitations. Although the sample size exceeded the minimum requirements and included professionals from different generations and practice environments, the use of the convenience sampling method may have limited the external validity and generalisability of the results. Furthermore, while the cross-sectional design was suitable for assessing validity and internal consistency, a conclusion regarding temporal stability cannot be drawn because it does not permit longitudinal analyses, such as test–retest reliability, which would offer evidence of the instrument’s stability over time.

The study was also conducted in a single geographic area, which, despite its size, may not reflect the diversity of professional contexts across the country. Additionally, the use of paper-based questionnaires distributed through nurse managers may have introduced response bias, particularly social desirability bias.

Another limitation concerns the absence of measurement invariance testing across relevant subgroups. As a result, it is unclear whether the factor structure and item functioning of the Portuguese NPI are consistent across different groups, which may limit the generalisability of the results.

Potential self-report bias, inherent to questionnaire-based assessments of professionalism, may also have influenced participants’ responses. Moreover, incomplete questionnaires were excluded without conducting sensitivity analyses, which could have clarified the impact of missing data handling on the results. Finally, the borderline RMSEA values observed in the confirmatory factor analysis should be acknowledged as a methodological limitation. Although these values suggest a marginal fit, they do not undermine the overall adequacy of the model, especially when considered alongside the favourable CFI, TLI, and SRMR indices.

## 5. Conclusions

This study provides a validated and reliable Portuguese version of the NPI, contributing to the availability of tools for assessing nurses’ professionalism in Portugal.

The confirmation of the five-factor structure and high internal consistency make the NPI a solid tool for the systematic assessment of professional behaviours and attitudes. The observed associations with structural empowerment and psychological safety further support its construct validity, although these correlations were generally weak yet statistically significant. Given these findings, the Portuguese version of the NPI can be a valuable resource for research, education, and nursing management. Nonetheless, its utilisation should account for the study’s methodological limitations, including convenience sampling, geographic restriction, a cross-sectional design, the absence of longitudinal testing, and borderline RMSEA values. Future research should investigate measurement invariance across subgroups, assess test–retest reliability, and explore the instrument’s applicability in diverse clinical and organisational settings. Overall, the present study endorses the NPI as a promising tool for assessing nurses’ professionalism in the Portuguese context.

## Figures and Tables

**Table 1:** Sociodemographic, professional, and academic characteristics of the participants (*n* = 684).

Variable	
Age (years) Mean; Std. Dev.	40.3; 9.4
Gender *n* (%)	
Female	553 (80.8)
Male	131 (19.2)
Education *n* (%)	
Bachelor’s degree	576 (84.2)
Master’s degree	108 (15.8)
Professional category *n* (%)	
Nurse	526 (76.9)
Specialist nurse	151 (22.1)
Nurse manager	7 (1.0)
Professional title *n* (%)	
Nurse	433 (63.3)
Specialist nurse	251 (36.7)
Nursing Specialisation *n* (%)	
Rehabilitation Nursing	78 (31.1)
Medical–Surgical Nursing	56 (22.3)
Child and Pediatric Health Nursing	34 (13.6)
Community Nursing	24 (9.6)
Maternal and Obstetric Nursing	23 (9.2)
Medical–Surgical Nursing in Critical Care	13 (5.2)
Medical–Surgical Nursing in Palliative Care	11 (4.4)
Community and Public Health Nursing	6 (2.4)
Psychiatry and Mental Health Nursing	3 (1.2)
Community and Family Health Nursing	2 (0.8)
Medical–Surgical Nursing in Perioperative Care	1 (0.4)
Work Department *n* (%)	
Medicine Department	223 (32.6)
Surgery Department	203 (29.7)
Intensive Care Department	113 (16.5)
Woman and Child Department	77 (11.3)
Emergency Department	68 (9.9)
Time of professional practice (years) Mean; Std. Dev	17.0 (9.3)
Time of professional practice in the service (years) Mean; Std. Dev	11.0 (9.0)

Std. Dev.—Standard deviation.

**Table 2:** Average Variance Extracted and internal consistency in the initial model of the subscales of the Portuguese version of the NPI (*n* = 684).

Subscales of the Portuguese Version of the NPI	Average Variance Extracted (AVE)	Internal Consistency		
		Composite Reliability	McDonald’s Omega	Cronbach’s Alpha
Accountability	0.48	0.82	0.83	0.80
Self-improvement	0.53	0.88	0.99	0.85
Professional Attitude	0.46	0.75	0.79	0.71
Advancement of the Nursing Profession	0.62	0.81	0.81	0.81
Professional Membership	0.78	0.86	0.89	0.82

**Table 3:** Average Variance Extracted and internal consistency in the final model of the subscales of the Portuguese version of the NPI (*n* = 684).

Subscales of the Portuguese Version of the NPI	Average Variance Extracted (AVE)	Internal Consistency		
		Composite Reliability	McDonald’s Omega	Cronbach’s Alpha
Accountability	0.48	0.82	0.83	0.80
Self-improvement	0.53	0.88	0.99	0.85
Professional Attitude	0.52	0.77	0.81	0.73
Advancement of the Nursing Profession	0.62	0.81	0.81	0.81
Professional Membership	0.78	0.86	0.89	0.82

**Table 4:** Factor loadings of the items in their respective constructs in the final model (*n* = 684).

Subscales of the Portuguese Version of the NPI	Item	Factor Loading
Accountability	Item 1	0.72
	Item 2	0.67
	Item 3	0.67
	Item 4	0.74
	Item 5	0.69
	Item 6	0.64
	Item 7	0.73
	Item 8	0.69
Self-improvement	Item 9	0.83
	Item 10	0.81
	Item 11	0.59
	Item 12	0.69
	Item 13	0.65
	Item 14	0.76
	Item 15	0.72
	Item 16	0.74
Professional Attitude	Item 17	0.75
	Item 19	0.68
	Item 20	0.75
	Item 21	0.71
Advancement of the Nursing Profession	Item 22	0.64
	Item 23	0.83
	Item 24	0.85
	Item 25	0.83
Professional Membership	Item 26	0.91
	Item 27	0.93
	Item 28	0.80

**Table 5:** Spearman correlation coefficient and respective *p*-value between the NPI subscales, the CWEQ-II dimensions, and TPS (*n* = 684).

	Accountability	Self-Improvement	Professional Attitude	Advancement of the Nursing Profession	Professional Membership
CWEQ-II Opportunity	0.157	0.202	0.170	0.120	0.164
*p*-value	<0.0001	<0.0001	<0.0001	0.0016	<0.0001
CWEQ-II Information	0.183	0.271	0.237	0.175	0.090
*p*-value	<0.0001	<0.0001	<0.0001	<0.0001	0.0192
CWEQ-II Support	0.141	0.212	0.205	0.137	0.111
*p*-value	0.0002	<0.0001	<0.0001	0.0003	0.0035
CWEQ-II Resources	0.124	0.228	0.170	0.100	0.079
*p*-value	0.0011	<0.0001	<0.0001	0.0090	0.0389
CWEQ-II Formal Power	0.133	0.208	0.170	0.092	0.063
*p*-value	0.0005	<0.0001	<0.0001	0.0161	0.1000
CWEQ-II Informal Power	0.171	0.299	0.259	0.290	0.209
*p*-value	<0.0001	<0.0001	<0.0001	<0.0001	<0.0001
TPS	0.162	0.185	0.168	0.146	0.136
*p*-value	<0.0001	<0.0001	<0.0001	0.0001	0.0004

## Data Availability

The data presented in this study are available on request from the corresponding author. The data are not publicly available due to privacy or ethical restrictions.

## Public Involvement Statement

No public involvement in any aspect of this research.

## Guidelines and Standards Statement

This manuscript was prepared in accordance with COSMIN for methodological research.

## Abbreviations

The following abbreviations are used in this manuscript:

AVE	Average Variance Extracted
CFI	Comparative Fit Index
CWEQ-II	Conditions of Work Effectiveness Questionnaire II
CI	Confidence Interval
CFA	Confirmatory Factor Analysis
NPI	Nurses’ Professionalism Inventory
RMSEA	Root Mean Square Error of Approximation
SEE-NP	Scale for the Environment Evaluation of Professional Nursing Practice
SRMR	Standardized Root Mean Square Residual
TPS	Team Psychological Safety
TLI	Tucker–Lewis Index
WLSMV	Weighted Least Squares Means and Variances
